# Research on Bearing Fault Diagnosis Method Based on an Adaptive Anti-Noise Network under Long Time Series

**DOI:** 10.3390/s20247031

**Published:** 2020-12-08

**Authors:** Changdong Wang, Hongchun Sun, Rong Zhao, Xu Cao

**Affiliations:** 1 School of Mechanical Engineering and Automation, Northeastern University, Shenyang 110819, China; 1870188@stu.neu.edu.cn (C.W.); caoxu.zai@foxmail.com (X.C.); 2 Key Laboratory of Vibration and Control of Aero-Propulsion Systems of Ministry of Education, Northeastern University, Shenyang 110819, China; 3 College of Sciences, Northeastern University, Shenyang 110819, China; 1800126@stu.neu.edu.cn

**Keywords:** bearing fault diagnosis, longer time series, one-dimensional convolutional network, hyperparameter adaptation, anti-noise

## Abstract

In the era of big data, longer time series fault signals will not only be easy to copy and store, but also reduce the labor cost of manual labeling, which can better meet the needs of industrial big data. Aiming to effectively extract the key classification information from a longer time series of bearing vibration signals and achieve high diagnostic accuracy under noise and different load conditions. The one-dimensional adaptive long sequence convolutional network (ALSCN) is proposed. ALSCN can better extract features directly from high-dimensional original signals without manually extracting features and relying on expert knowledge. By adding two improved multi-scale modules, ALSCN can not only extract important features efficiently from noise signals, but also alleviate the problem of losing key information due to continuous down-sampling. Moreover, a Bayesian optimization algorithm is constructed to automatically find the best combination of hyperparameters in ALSCN. Based on two bearing data sets, the model is compared with traditional model such as SVM and deep learning models such as convolutional neural networks (CNN) et al. The results prove that ALSCN has a higher diagnostic accuracy rate on 5120-dimensional sequences under −5 signal to noise ratio (SNR) with better generalization.

## 1. Introduction

Rolling bearings are prevalent components in rotating machinery for modern industrial applications [1]. Moreover, the health status of rolling bearings has a huge impact on the performance stability and service life of the entire mechanical equipment [2]. What needs more attention is that their failure is one of the most frequent reasons for machine breakdown [3]. Therefore, it is completely necessary to make fault diagnosis for rolling bearings. Nevertheless, the field of mechanical fault diagnosis has entered the era of big data [4]. As the number and types of acquisition equipment are increasing, the collected signals are growing exponentially. This puts forward higher requirements for the signal in terms of replication, storage, and effective compression. Literature [5] referred that longer signal samples in a suitable range can save storage costs and time. At present, most of the literatures [6,7,8,9] generally used 2048 or shorter lengths sample lengths when studying bearing fault diagnosis to achieve higher accuracy. However, they only considered the sample length that satisfies the normal operation cycle of the bearing and ignored the problem of data storage and transmission under big data.

Traditional fault diagnosis method such as support vector machines (SVM) is often unable to automatically extract high-level features of bearing data and require too much prior knowledge. Moreover, the selection of features directly affects the effect of fault diagnosis, and it is difficult to apply to massive data [10]. One of the characteristics of big data is to meet real-time. However, the traditional feature extraction process is computationally intensive and hinders the real-time use of the monitoring program [11]. The advent of deep learning theories has reformed intelligent fault diagnosis in further releasing the artificial assistance since the 2010s. Correspondingly, the end-to-end diagnosis procedure is encouraged to construct [12]. The end-to-end method establishes a direct connection between the vibration signal and the type of fault by omitting the feature extraction process. Therefore, through deep learning method, the automation and intelligence of bearing fault diagnosis are better promoted [13]. In order to reduce the process of manual feature extraction, some end-to-end deep learning models have been proposed one after another [14]. Gao proposed a gearbox bearing fault diagnosis method based on self-reference adaptive noise cancellation technology (SANC) and one-dimensional convolutional neural network (1D-CNN) [15]. Based on the model of RNN [16], Liu proposed a new bearing fault diagnosis method in the form of an auto-encoder. At the same time, there are some bearing fault diagnosis algorithms based on deep belief net (DBN) [17], deep CNN [18,19] and back propagation neural network (BPNN) [20]. After experimental verification, the accuracy of fault diagnosis of these models is ideal. However, ruled fully connected deep learning networks will be limited when solving more complex problems. Because their network bias and weight parameters will increase exponentially with the increase of the model layer. It will lead to overfitting and gradient disappearance. Furthermore, almost all these models need to increase the number of layers to make themselves perform better in the classification of longer signals [21]. More importantly, when the amount of data reaches a very large scale, the storage of short time series has to match more information. It will increase the burden on the hardware. Moreover, blindly upgrading hardware can also lead to extremely high costs. In order to better implement engineering applications, feature extraction under noise is also a challenge that must be faced [22]. Therefore, it is of great significance to propose an anti-noise network model that considers both efficient storage and accurate fault diagnosis under big data.

Although CNN used as the backbone network in this paper also has the above problems. However, the research in this article is dedicated to improving these issues. CNN [23] has been applied because of some attractive advantages, such as displacement invariance and weight distribution [24]. Not only that, but its hierarchical feature visualization function is also easier to study than RNNs that contain numerous hidden layers. All in all, CNN has been proved successful in many fields [25,26,27,28].

Based on the thinking, this paper proposes a model named adaptive long sequence convolutional network (ALSCN) which can balance diagnosis accuracy and big data requirements without deepening the model [29]. The main contributions of this literature are summarized as follows.

1. An end-to-end ALSCN is proposed to bearing fault diagnosis which performs well under strong noise and different loads environment simultaneously. In addition, ALSCN can directly act on longer original signals, thereby reducing the workload of manual signal preprocessing.
2. The multi-filter-layer based on improved atrous spatial pyramid pooling (ASPP) [30] is developed to preserve the spatial correlation of longer raw fault signal under noise, and the multi-scale pooling module is constructed to compensate for the loss.
3. The Bayesian optimization algorithm are applied to optimize the hyperparameters for reducing the time of manual parameter adjustment. Furthermore, after removing the fully connected layer, ALSCN has an ideal cost in parameter calculation.
4. Firstly, by visualizing the feature learning process between different layers of the network, the internal mechanism is explored. Then, the different results which are caused by the different order of modules and pooling operations are discussed and explained. Finally, the best structure for the ALSCN is given.
5. Based on the bearing fault data set of Case Western Reserve University (CWRU) and the mechanical fault prevention technology (MFPT), the ALSCN model proposed outperforms SVM, CNN, RNN, DBN, and BP neural network models.

The rest of the paper is organized as follows. In Section 2, the related theories of one-dimensional convolutional networks, receptive fields, dilated convolution, and Bayesian optimization are elucidated. Section 3 introduces the model structure proposed in this paper, and the specific implementation process of the improved multi-scale feature extraction module as well as multi-scale pooling module. The relevant model training and test results are discussed, and analysis of experimental results are presented in Section 4. Section 5 summarizes the full text and put forward the theme of future research.

## 2. Related Theories

### 2.1. One-Dimensional Convolutional Network

**Figure 1:**
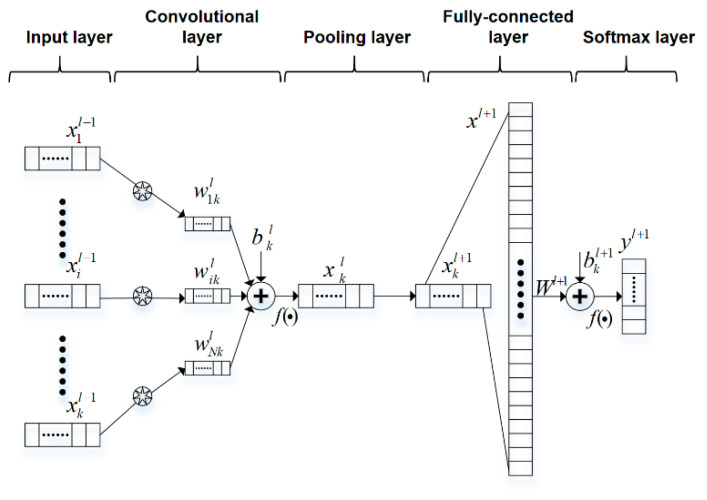
The principle of 1-D convolutional neural networks.

A typical convolutional neural network structure is shown in Figure 1, which usually includes an input layer, a convolutional layer, a pooling layer, a fully connected layer, and an output layer. When using for classification, CNN are usually divided into two main stages: using convolutional layers to extract features and mapping these features into categories through fully connected layers. Different from traditional neural networks, the feature extractor of convolutional neural networks is composed of convolutional layers and pooling layers. The weight sharing and local connection of the convolution kernel greatly reduce the parameters of the model and lower the complexity of the convolutional neural network and the risk of overfitting.

### 2.2. Receptive Field

The receptive field is defined as the area where the convolutional neural network features can see the input image. The larger the receptive field is, the farther distance of characteristic relationship between objects that network can learn. In addition, the deeper layer of a network is, the greater range that network can perceive. For long time sequences, the distribution of key classification points is more random and changeable, and the important classification information which is captured in the deep neural network will also be more abstract. Therefore, for capturing key classification features on long time sequences more effectively, expanding the receptive field of the network is a wonderful method. The calculation formula of the receptive field can be expressed as

(1) $$r_{n}=r_{n-1}+\left(k-1\right)\times \prod_{I=1}^{n-1}S_{i}$$

where $r_{n}$ is the receptive field of the layer, $r_{n-1}$ is the receptive field of the previous layer, $S_{i}$ is the step size of the convolution or pooling at the layer i, and k is the size of the convolution kernel.

At present, there are two main methods for increasing the receptive field [31]. (1) Building a network with more layers as well as add a convolution filter to achieve linear increase in the receptive field. (2) Using down-sampling to increase the receptive field by pooling and stacking different layers of convolutional neural networks.

### 2.3. Dilated Convolution

**Figure 2:**
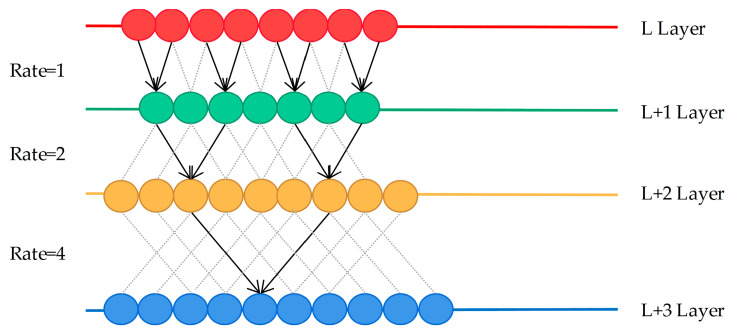
The working principle of dilated convolution.

In the field of bearing fault diagnosis, the convolutional network will cause insufficient feature extraction due to continuous pooling operations. Obviously, it will affect the accuracy of diagnosis. To overcome the above problem, the concept of dilated convolution is introduced in this section. A parameter called dilated rate is presented by the dilated convolution to define the distance between the values. In dilated convolution, the elements of the convolution kernel are spaced, and the size of the space depends on the dilated rate. A principle of expanding the receptive field on a one-dimensional vector is shown in Figure 2.

In Figure 2, straight lines of different colors are used to represent the one-dimensional vectors of different layers. When the dilated rate is one, it is similar to ordinary convolution sampling without intervals. When the dilated rate is two, it means that every column of samples on the L + 1 layer are convolved with the convolution kernel. By analogy, every three columns are sampled at equal intervals and then convolved at the next layer when the dilated rate is four.

The calculation method of the dilated convolution is the equal interval sampling, and the convolution result obtained by one layer always comes from an independent set of the previous layer. Hence, it always causes a certain problem of information loss. Moreover, the dilated convolution samples the input signal sparsely. This makes no correlation between the key features of the long-time sequences obtained by convolution, thereby affecting the classification result. Thus, the spatial pyramid pool and dilated convolution are introduced here to solve the above problem. The spatial pyramid pool was first introduced to CNN by He et al. [32] to meet the fixed-length requirements of classification neurons for classification recognition. The working principle of the spatial pooling feature pyramid is to implement parallel convolution of different sampling rates on the same input, and then perform feature fusion.

### 2.4. Bayesian Optimization Algorithm

The computational cost of CNN is relatively high, and it may take plenty of time to train on traditional platforms. Moreover, no CNN model can best generalize all data sets [33]. The method of manually adjusting parameters is inefficient and time-consuming in actual engineering application. Therefore, accurate and appropriate hyperparameter adjustment is a normal state in the fault diagnosis of industrial applications. As an important hyperparameter in CNN, learning rate can directly affect the quality of classification results. Too large learning rate may lead to poor convergence, and too small learning rate may lead to slow convergence. Thence, when applying CNN to a new data set, an appropriate learning rate must be selected first to meet the needs of better practical applications. Bayesian optimization [34] as an efficient global optimization algorithm can solve the above problems well. The Bayesian optimization process has three main parts.

1. Given the objective function, random sampling is performed in the parameter space.
2. Obtain the initial objective function distribution, and then continuously search for the optimal solution of the objective function based on historical information.
3. Iterate continuously until the distribution fitted by the sampling points is roughly the same as the true objective function. In order to fit the relationship between parameter selection and objective function more comprehensively, Bayesian optimization puts forward the idea of probabilistic surrogate model. Bayesian optimization consists of two parts, the probabilistic surrogate model and the acquisition function.

The update of the probabilistic surrogate model is determined by

(2) $$p\left(f|D_{1:t}\right)=\frac{p\left(D_{1:t}|f\right)p\left(f\right)}{p\left(D_{1:t}\right)}$$

where f is the unknown objective function, $D_{1:t}$ is the collection of collected sample points, $p\left(D_{1:t}|f\right)$ is the likelihood distribution of y, p(f) is the prior probability distribution model of f, $p\left(D_{1:t}\right)$ is the marginal likelihood distribution of marginalized f, $p\left(f|D_{1:t}\right)$ is the posterior probability distribution of f, namely, confidence of the unknown function after the prior probability distribution is modified.

Since the non-parametric probabilistic surrogate model is more flexible and easier to expand, it can better describe the unknown objective function. Among them, the Gaussian process with strong fitting performance is the most widely used [35].

**Figure 3:**
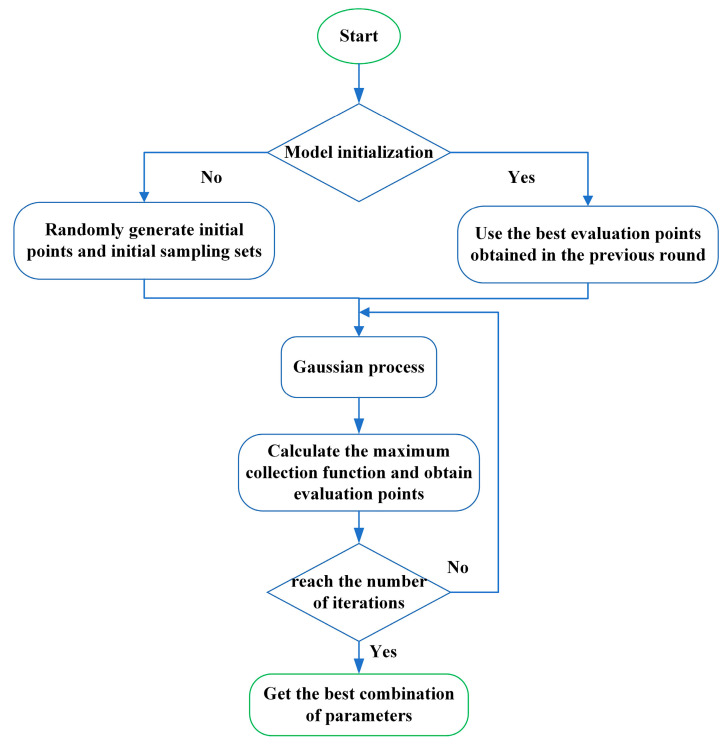
The process of Bayesian optimization.

The acquisition function is the basis for the targeted search for the next evaluation point in the parameter space. This article selected PI (Probability of Improvement) as the collection function. PI indicates that the next sample point collected may improve the possibility of the optimal objective function, as shown in the Equation (3).

(3) $$\alpha_{t}\left(x;D_{1:t}\right)=p\left(f\left(x\right)\leq v^{*}-\varepsilon \right)=\emptyset \left(\frac{v^{*}-\varepsilon -\mu_{t}\left(x\right)}{\sigma_{t}\left(x\right)}\right)$$

where $v^{*}$ is the optimal value of the current objective function, ∅(·) is the standard normal distribution cumulative density function, and ε is the balance parameter. By adjusting the size of ε, it is possible to realize the search for the optimum value globally. The Bayesian optimization process is given by the Figure 3.

## 3. Bearing Fault Diagnosis Method Based on ALSCN

**Figure 4:**
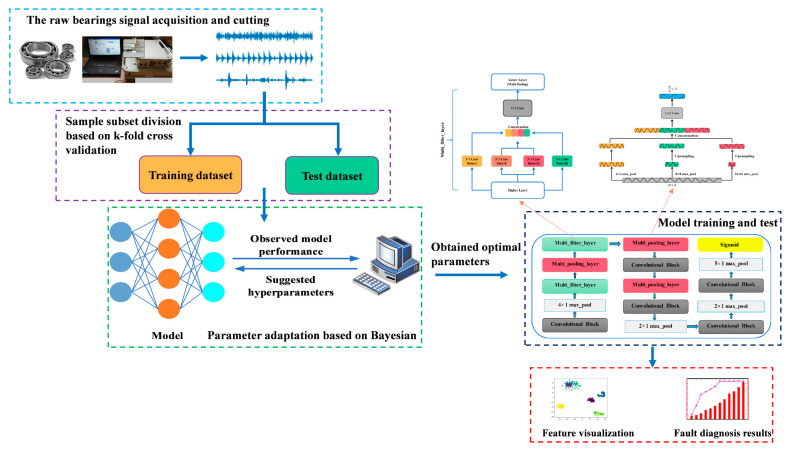
The bearing fault diagnosis method based on adaptive long sequence convolutional network (ALSCN).

The schematic architecture of bearing fault diagnosis method based on the proposed network is illustrated in Figure 4. The raw signal collected from a data acquisition system can be directly divided into training set and test set for use. Then, according to different working conditions, the hyperparameters will be selected adaptively. Then, the proposed network is implied to learn the feature of signal. The fault diagnosis result will be given at the end.

### 3.1. The Model Structure Proposed in This Paper

The structure of the convolutional block consists of a convolutional layer, batch normalization (BN) and rectified linear unit (ReLU). In the convolutional layer, the local area of the input is convolved through the kernel of the filter. Subsequently, under the action of the activation unit, the output features are effectively generated. BN is used behind the convolutional layer to make the training time shorter and reduce the offset of the internal covariance. In recent years, ReLU as a vital part is widely used in the example of activation unit. Because ReLU can increase the representation ability of the network and make the learned features easier to divide.

**Figure 5:**
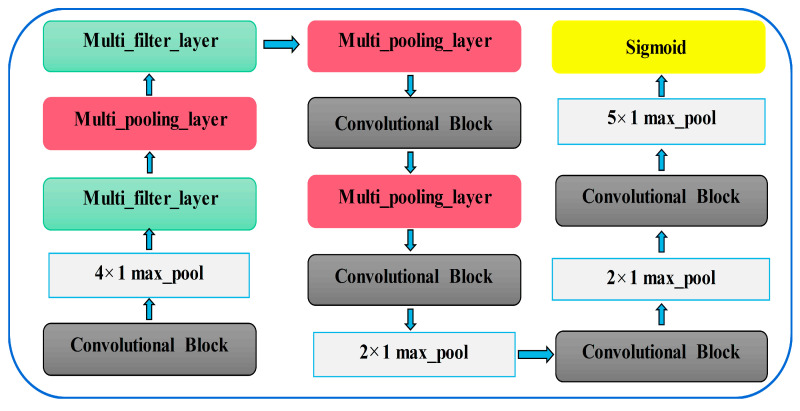
The structure of the ALSCN model.

The model structure proposed in this paper is shown in Figure 5, which is composed of some filtering stages and one classification stage. Rely on CNN as the backbone network, the fully connected layer with numerous parameters is removed to reduce calculation costs. For convenience, multi-scale feature extraction module and multi-scale max pooling module are called multi-filter-layer and multi-pooling-layer, respectively. In addition, the multi-filter-layer based on the convolutions of different dilated rates and multi-pooling-layer are used to expand the receptive field while extracting multi-scale key information efficiently. Without any other transformation, the first convolutional block extracts feature from the input original N × 1 × 5120-dimensional fault signal. N is the number of batch processing, 1 represents the number of channels, and 5120 represents the data dimension. After that, the max pooling with kernel size of 4 and step of 4 are performed to improve the ability of model in learning features. Next, the key information of different scales is obtained at the same layer by combining the two modules in a certain order. Finally, normalized by the sigmoid function in the classification stage, a N × 7 × 1-dimensional vector is obtained. Details of each stage are given comprehensively in Table 1.

### 3.2. Multi-Scale Feature Extraction Module and Multi-Scale Max Pooling Module

Facing the disadvantage that the dilated convolution will cause the loss of information. Moreover, the problem of weak spatial correlation between the long time series features extracted using standard convolution. This paper improves the attentive spatial pyramid pooling (ASPP) module and proposes a multi-scale max pooling module.

#### 3.2.1. Introduction of Multi-Scale Feature Extraction Module

**Figure 6:**
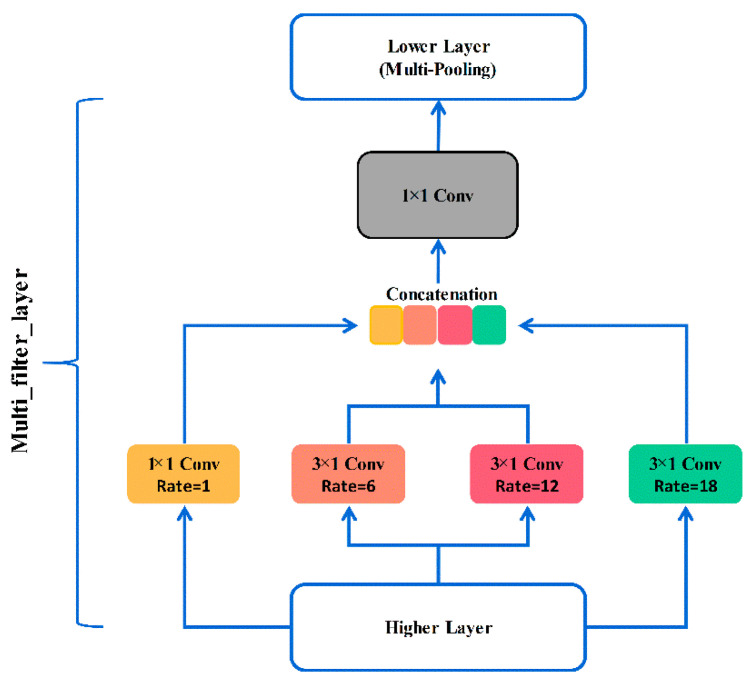
The structure of multi-scale feature extraction module.

Due to the excessive number of layers of the inception series networks, the network will be difficult to train or even degraded, and it is also prone to overfitting [36]. However, the idea of multi-scale feature extraction makes some bearing fault diagnosis methods have stronger generalization performance under noise. Inspired by Inception [37] series networks, the multi-scale feature extraction module removes the pooling branch to preserve the highest response of data points better. Further, three ordinary 1 × 1 convolutions in the traditional CNN are replaced by different dilated convolutions. In this way, the key features of the different receptive fields can be obtained in the same layer. As can be seen from Figure 6, the module consists of three parts. The first part is multi-branch convolution. Convolution kernels of different dilated rates are used to capture the feature relationship at different intervals. Moreover, the convolution features of different branches will be fused in next part. Finally, the 1 × 1 convolution is introduced to perform convolution processing. The multi-scale feature extraction module can make the network of this article not rely too much on some local features to enhance the generalization. Note that this is completely different from the principle of dropout, where the activation value of a certain neuron stops working with a certain probability. The following is the detailed process.

The previous layer of the module is a convolution block with a convolution kernel size of one and a step size of one. Then, the output of the convolution block is used as the input of the module. After that, one dilated convolution (convolution kernel: 1 × 1, dilated rate: 1) and three dilated convolutions (convolution kernel: 3 × 1, dilated rate: 6, 12, 18) are parallel to extract features widely. Note that, the value of Batch is set in advance, and the 1 × D features output from the four branches are combined according to the channel to obtain 4 × D features. Thus, the above operation can make up for the loss of key information in the long time sequences. Then the features are processed with a 1 × 1 convolution to obtain a 1 × D feature as the output of the module. The max pooling is performed to preserve the main features at last.

The procedure for the multi-scale feature extraction can be stated as Algorithm 1.
**Algorithm 1** Multi-scale feature extraction moduleInput: features $a_{i}\in R^{batch\_size\times m\times d}$, i = 1, 2…N; Output: features $a_{i}^{\prime }\in R^{batch\_size\times m\times \frac{d}{4}}$, i = 1, 2…N.Perform four convolution operations on the input vector $a_{i}$ respectively, where the size of the convolution kernels is 1 × 1, 3 × 1, 3 × 1 and 3 × 1. Moreover, the step size is 1, the dilated rates are 6, 12, 18, separately. To obtain the convoluted feature vectors: $conv_{1},conv_{2},conv_{3},conv_{4}\in R^{batch\_size\times m\times \frac{d}{4}}$.Splice $conv_{i},\;i=1,2,3,4$ according to the channel to get the feature vector $conv^{\prime }\in R^{batch\_size\times m\times d}$.Perform 1 × 1 convolution operation on $conv^{\prime }\in R^{batch\_size\times m\times d}$ to compress the feature dimensions to obtain the feature vector $conv^{\prime \prime }\in R^{batch\_size\times m\times \frac{d}{4}}$.Perform a max pooling operation of 2 × 2 with a step size of 2 on $conv^{\prime \prime }$, after ReLU activation and BN operation, get the final feature vector $a_{i}^{\prime }\in R^{batch\_size\times m\times \frac{d}{4}}$, i = 1, 2…N.


#### 3.2.2. Introduction of Multi-Scale Max Pooling Module

**Figure 7:**
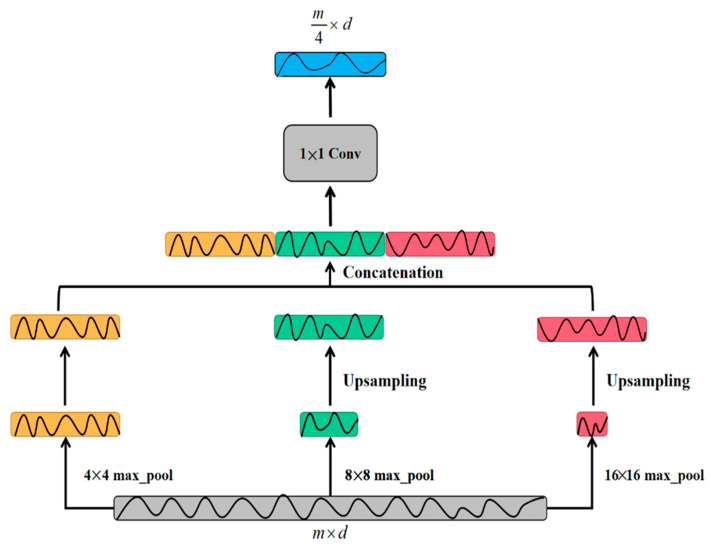
The structure of multi-scale pooling module.

This paper proposes a multi-scale max pooling module to reduce information loss in max pooling. As given in Figure 7, the working principle of the module is divided into four parts. First of all, the three pooling branches are parallelized, and then the max pooling operations of 4 × 4, 8 × 8, and 16 × 16 are performed respectively to obtain 1/4, 1/8, and 1/16 of the original signal feature vector. By doing this, the module excellently preserves the spatial correlation between data points at different intervals in the original signal. Secondly, the two features with smaller resolution are changed to 1/4 of the original features by using the nearest neighbor interpolation method. In next step, the various features are combined according to the channel in the third part. Finally, the convolution of 1 × 1 is used to change the dimension, and the obtained features containing multi-scale sampling information are used as the output of the module.

The procedure for the multi-scale max pooling can be stated as Algorithm 2.
**Algorithm 2** Multi-scale max pooling moduleInput: features $b_{i}\in R^{batch\_size\times m\times d}$, i = 1, 2…N; Output: features $b_{i}^{\prime }\in R^{batch\_size\times m\times \frac{d}{4}}$, i = 1, 2…N.Perform max pooling operation with the size of 4 × 4, 8 × 8, 16 × 16, and the step size of 4, 8, 16 on the input vector $b_{i}$, respectively, to obtain the pooled feature vector: $pooling_{1}\in R^{batch\_size\times \frac{m}{4}\times d}$, $pooling_{2}\in R^{batch\_size\times \frac{m}{8}\times d}$, $pooling_{3}\in R^{batch\_size\times \frac{m}{16}\times d}$.Double upsampling $pooling_{2}$ to get the feature vector $pooling_{2}^{\prime }\in R^{batch\_size\times \frac{m}{4}\times d}$ by using nearest neighbor linear interpolation.Four times upsampling $pooling_{3}$ to get the feature vector $pooling_{3}^{\prime }\in R^{batch\_size\times \frac{m}{4}\times d}$ by using nearest neighbor linear interpolation.Splice $pooling_{1}$, $pooling_{2}^{\prime }$, $pooling_{3}^{\prime }$ according to the channel to get the feature vector $pooling^{\prime \prime }\in R^{batch\_size\times m\times \frac{d}{4}}$.Perform the convolution operation of 1 × 1 on $pooling^{\prime \prime }$. Afterward, merge features, perform ReLU activation and BN operations to finally obtain the output vector $b_{i}^{\prime }\in R^{batch\_size\times m\times \frac{d}{4}}$, i = 1, 2…N.


## 4. Experimental Verification

In this paper, network training and verification of the results are carried out based on the CWRU bearing fault data set and MFPT bearing fault data set. In addition, regularization and batch normalization are used during the training process to ensure the stability of the model and prevent the problem of gradient explosion. The main evaluation index to measure the quality of the model is accuracy, which is defined as follows:

(4) $$ACC=\frac{TP+TN}{TP+TN+FP+FN}$$

where *TP* represents the number of positive classes predicted as positive classes, *FN* indicates the number of positive classes predicted as negative classes, *FP* is the number of negative classes predicted as positive classes, and *TN* is the number of negative classes predicted as negative classes. 

The main parameters of the network are shown in Table 2. The “/” means no parameters. According to the Bayesian optimization the batch number of training is selected as 64, the epoch is 200 in the model and the learning rate is selected 0.001. For fair comparison the hyperparameters of other models are also adjusted to the optimal conditions by Bayesian optimization. For the selection of BPNN, RNN, and DBN structure, this paper referred to the methods in literature [38,39,40], respectively. At the same time, considering the dimensions of data input and the layer structure, the final structure selections are as follows: (1) The structure of BPNN is selected as 5120-500-7. The learning rate, momentum, and iteration number are 0.1, 0.9, and 400, respectively. (2) The RNN is composed of three LSTM layers which each layer has 50 hidden layers, and the batch size is 60. (3) The structure of DBN is given as 1280-320-80-20-7. The learning rate and iteration number are same as parameters of BPNN, and the momentum is 0.8. (4) In SVM, RBF kernel is applied, penalty coefficient and gamma value are set to 30 and 0.001. This paper also builds a one-dimensional CNN with fully connected layer and ordinary convolution. In order to make a fair comparison, the main parameters are the same as ALSCN. Moreover, all tests were performed on computers based on Linux system Cuda 9.0.176, Cudnn-major 7 and Python3.6, Pytorch1.1.

### 4.1. Experimental Data

**Figure 8:**
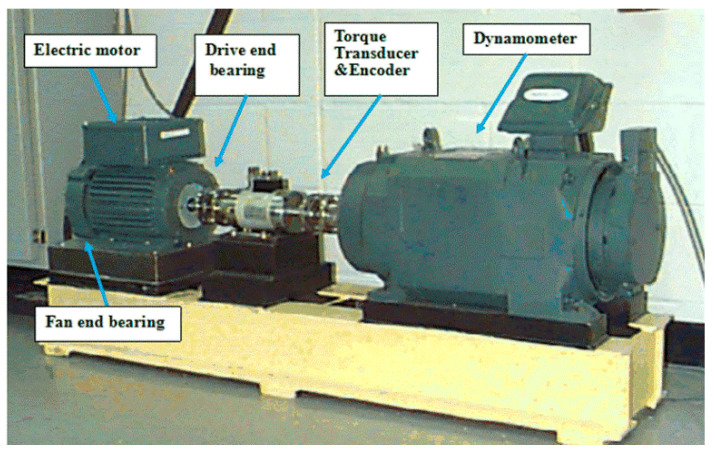
Experiment equipment provided by Case Western Reserve University (CWRU).

The data set from Case Western Reserve University bearing data center [41] is analyzed. The experimental equipment consists of a two-horsepower motor, a torque sensor, and a dynamometer, as shown in Figure 8. The accelerometer is used to collect vibration signals from three different positions: driver end, fan end, and base. In this experiment, the bearing damage is a single-point damage made by electrical discharge machining, and the fault data of the bearing at the drive end was mainly used. Different bearing damage diameters will affect the vibration response of the motor/bearing system to varying degrees. In order to quantify this effect, this paper used the data of 0.18 mm and 0.36 mm faulty bearings for experimental verification, and the composed data set is shown in Table 3. In order to persuasively verify the generalization of the network, data set D is obtained by mixing A, B, and C. Each data set contains 6 failure types and one failure-free type, and each sample contains 5120 sampling points. The k-fold cross-validation method is used on the data set and the sample subsets are randomly generated according to the ratio of 8:2. This is to minimize the deviation between the training set/test set and the complete set, and make the training set/test set evenly include all types of failures. The choice of k value will largely affect bias and variance. In order to make the final test error have a reasonable variance and make full use of all data in the previous splitting process. The k was selected as 10. The specific sample number is shown in Table 3.

The different damage radius of the bearing load zone will also affect the vibration response of the motor/bearing system to varying degrees. In order to quantify this effect, this paper uses the 0.18 mm and 0.36 mm faulty bearing data for experimental verification.

### 4.2. Accuracy Comparison of Different Length Signals 

This paper conducted verification experiments based on data sets A, B, C, and D in order to verify the effect of signal length on accuracy. It can be outlined from Table 4 that the CNN can already achieve an average accuracy of 96.75% in realizing the classification of 1024-dimensional data. However, when the data dimension increases to 5120, the average classification accuracy of the CNN drops to 86.35%. This is also verified that the performance of one-dimensional CNN significantly decreased on longer signal classification.

### 4.3. Ablation Experiment

The ablation experiment is a method to study network performance by deleting part of the network. This section applied the ablation experiment on the data set D verify the influence of the innovative modules. No mod means that the two innovation modules are not added. Mod 1 indicates that the alone multi-scale feature extraction module is applied. Mod 2 means that the alone multi-scale pooling module is added. Mod 1 + 2 means that two modules are employed in model at the same time.

**Figure 9:**
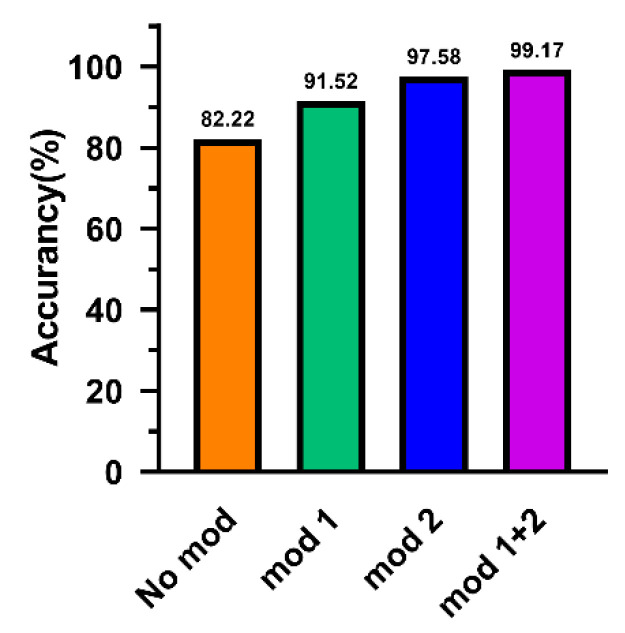
The results of ablation experiments.

As can be observed from Figure 9, without any modules, the accuracy on the 5120-dimention signal can only reach 82.22%. After adding the improved multi-scale feature extraction module individually, the classification accuracy rate promotes to 91.52%. Then, the alone multi-scale pooling module is applied in model, the classification accuracy reaches 97.58%. When the two modules are employed at the same time, the accuracy increases to 99.17%. This result proves that the innovative module achieves the purpose of effectively extracting features. It is more noteworthy that the data set used includes four types of loads. Therefore, the above results also reflect to a certain extent that ALSCN has a good adaptive ability in a mixed environment of multiple loads.

### 4.4. Model Optimal Structure Verification Experiment

In this section, in order to better evaluate the impact of innovation on model performance, the impact of the accuracy of improved modules in different network layers is mainly discussed. The reasons for the different accuracy caused by the different order of the pooling layers are explored. According to the experiment, the best sequence of modules and pooling layers are generated to provide reference for different tasks in the future. 

The accuracy of model fault diagnosis under three different module sequences is given in Table 5. It can be seen that the first model has the highest accuracy. In view of the relatively low accuracy of Model 2 and Model 3 in Table 5, this section will continue an in-depth analysis. In ALSCN, the previous layers extract shallow features to capture important classification information. At this time, the data features are sparse, and the relevant dependencies of multi-scale data as well as max response information should be more preferentially preserved. In deep layer, ALSCN should extract high-level semantic information; that is, a combination of shallow features and other information. The features at this time are dense and abstract. If the multi-scale convolution and response information are merged at a deeper level, the information flow will be confused to some extent. Therefore, the network cannot learn key features well.

At the same time, the accuracy verification experiments of pooling layers in different orders are also carried out. In Table 6, the highest diagnostic accuracy is achieved when max pooling of 2 × 1, 2 × 1, and 5 × 1 are used in stage 4, 5, and 6, respectively. It can be explained as that the network classification is equivalent to fitting the classification vector. As the network layer deepen, the vector length will gradually become shorter. At this time, 5 × 1 max pooling can better fit the short vector to produce the most effective gain to the final result.

**Figure 10:**
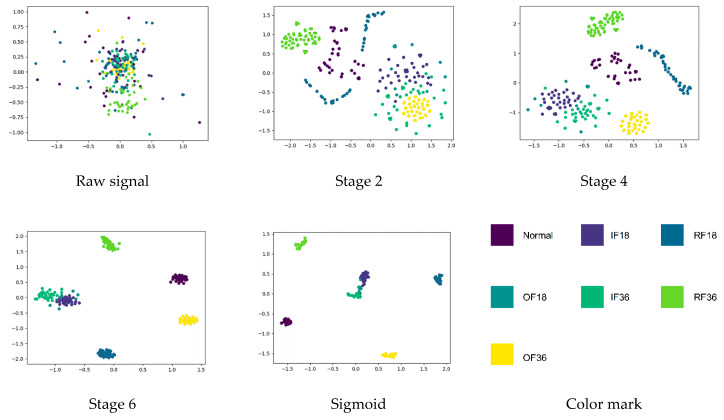
Learned feature visualization: feature representations extracted from raw signal, three stages, and sigmoid layer respectively.

After some thinking, the best structure of ALSCN can be obtained, as in Figure 4. In order to better explore the internal mechanism of ALSCN, this paper carried out internal feature visualization through t-SNE. By observing Figure 10, the distinction of features becomes more and more obvious as the network layers deepen. However, it is worth noting that, in the last layer of the network, OF18 and IF36 have a part of overlapping area. This means that the model has a certain error when distinguishing the outer ring fault of 0.18 mm and inner ring fault of 0.36 mm.

### 4.5. Model Time-Consuming Verification Experiment

**Figure 11:**
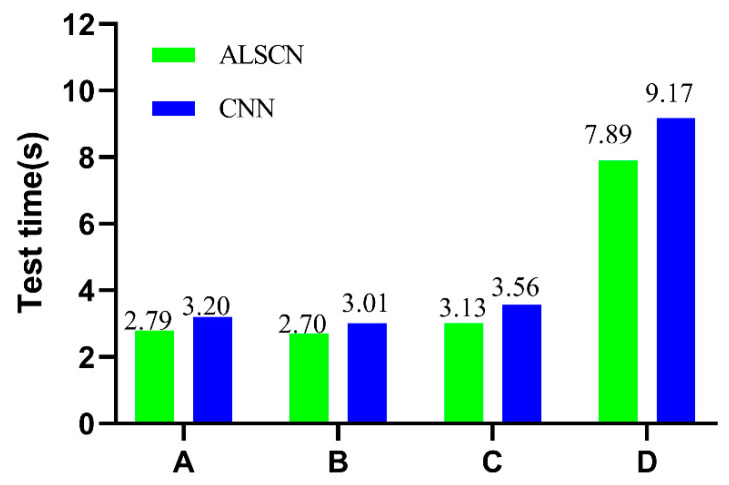
Comparison of test consuming between ALSCN and CNN.

ALSCN abandons the fully connected layer in the traditional CNN and achieves a high diagnostic accuracy. In order to verify its advantages in terms of calculation, the comparison model is selected as a one-dimensional CNN with a fully connected layer. The fully connected layer adopts a three-layer structure with dimensions of 400, 200, and 7, respectively. Considering the low computing power of embedded devices, the calculation comparison implemented on a computer with a CPU model of AMD Ryzen 5-4600U with Radeon Graphics 2.10 GHZ. The time-consuming comparison results of ALSCN and CNN on different data sets are revealed in Figure 11. The results show that ALSCN can consume less time than CNN during test to meet real-time requirement.

### 4.6. Network Performance Verification

#### 4.6.1. Robustness Verification

In a real application environment, noise will inevitably lead to a decrease in diagnostic accuracy. Noise was added to the signal to simulate the actual harsh environment. Moreover, the ability of ALSCN to resist noise is verified by comparison with traditional method and deep learning methods.

In statistics, the expression of the signal to noise ratio (SNR) is as follows:

(5) $$SNR=\frac{\sigma_{signal}^{2}}{\sigma_{noise}^{2}}$$

where $\sigma_{signal}$ is the variance of the signal, $\sigma_{noise}$ is the variance of the noise. In practical engineering applications, the expression of SNR is as follows:

(6) $$SNR\left(dB\right)=10log_{10}\left(\frac{P_{signal}}{P_{noise}}\right)=20log_{10}\left(\frac{A_{signal}}{A_{noise}}\right)$$

where $P_{signal}$ is the signal power, $P_{noise}$ is the noise power, $A_{signal}$ is the variance of the signal, and $A_{noise}$ is the variance of the noise.

**Figure 12:**
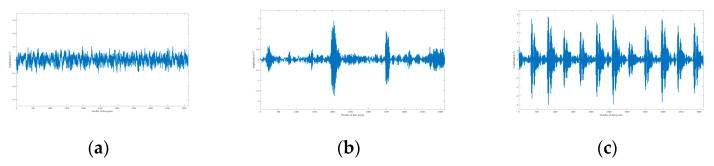
Simulation signals combined without noise. (**a**) Normal; (**b**) roller fault; (**c**) outer ring fault at 6:00 position relative to the load center.

**Figure 13:**
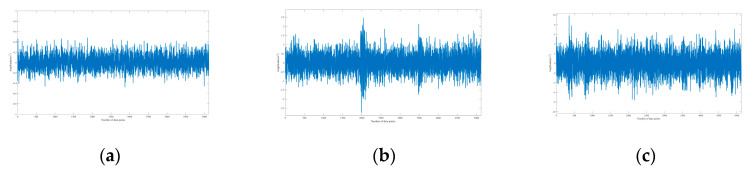
Simulation signals combined with noise (SNR = −5 dB). (**a**) Normal; (**b**) roller fault; (**c**) outer ring fault at 6:00 position relative to the load center.

The signal with a SNR between −14 dB and 0 dB is defined as a weak signal. According to Equations (5) and (6), the Gaussian noise is added to all samples to synthesize composite signals with different SNR. As given in Figure 12, the simulation signals without noise can reflect the different health status of the bearing, and the signals combined with noise (SNR = −5 dB) are shown in Figure 13.

**Figure 14:**
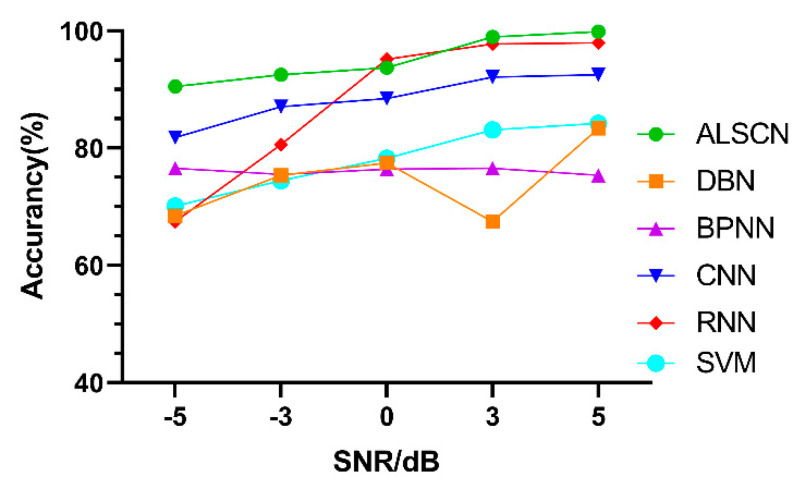
Test accuracy of different models under different signal to noise ratio (SNR) on CWRU.

Afterwards, based on the data set D, a comparison experiment is conducted with the accuracy of other models. The comparison results are demonstrated in Figure 14.

It can be seen from Table 7 that, the ALSCN obtains the highest test accuracy under strong noise (SNR = −5 dB). This is because the constructed two modules can more effectively extract the maximum response information from the signal under strong noise.

#### 4.6.2. Generalization Verification

This section conducted experiments on the four data sets to further prove the generalization of ALSCN under different loads. According to the confidence level of 95, the corresponding confidence interval is given to better rule out the contingency of experiment. The confidence interval calculation equation is as follows.

(7) $$\left[\mu -c\frac{\sigma }{\sqrt{n}},\mu +c\frac{\sigma }{\sqrt{n}}\right]$$

where μ is the sample mean, σ is the standard deviation, and n is the number of samples in one experiment, the value of c is 1.96.

**Figure 15:**
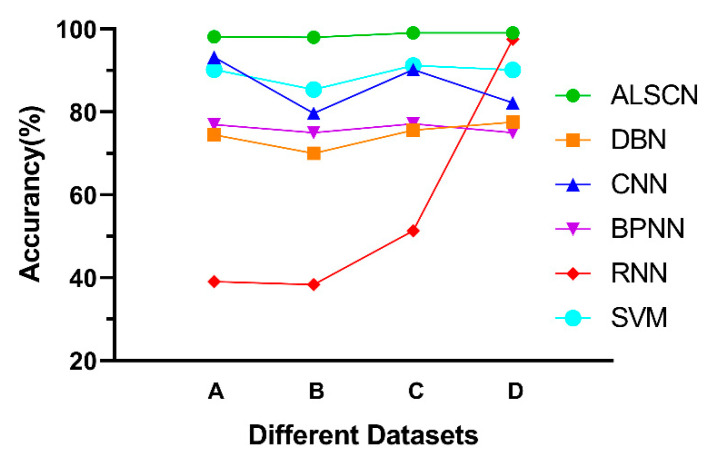
Comparison of test accuracy of different networks.

By observing Figure 15 and Table 8, the average accuracy of the ALSCN on four data sets is 98.62% through calculation. The most representative SVM in the traditional method achieves an average accuracy of 89.28% on long time series. In deep learning methods, the average accuracy of CNN, DBN, BPNN, and RNN are 86.35%, 74.45%, 76.03%, and 56.60%, respectively. Based on the fault diagnosis of a longer time sequence, ALSCN has achieved a clear advantage in accuracy. It is worth discussing that, the sample size of data set D is relatively large, the ALSCN has a slight improvement in accuracy compared with RNN. Nevertheless, the performance of RNN on data sets A, B, and C is obviously poor in the case of a small sample size. Therefore, it can be seen that the ALSCN is more advantageous in the case of few samples.

**Figure 16:**
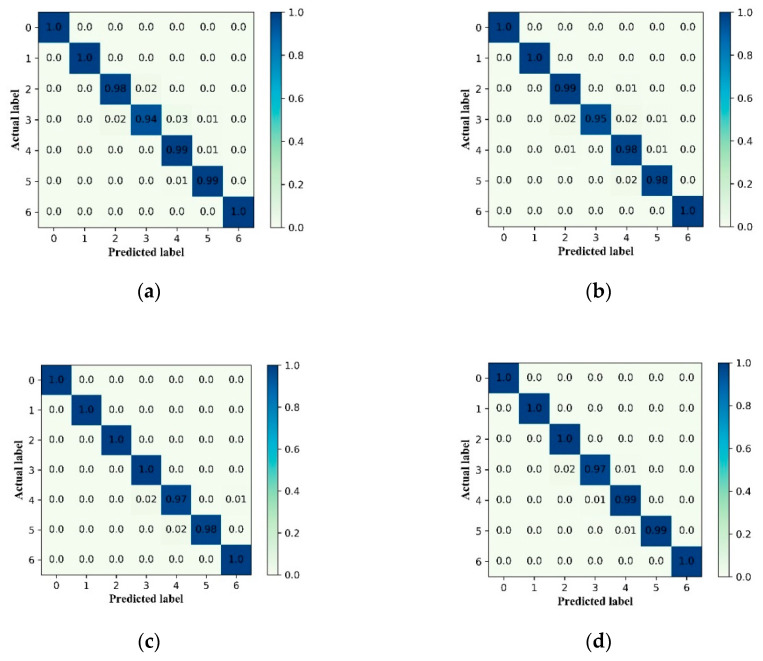
The confusion matrix of test process using the ALSCN on four data sets: (**a**) Data set A, (**b**) Data set B, (**c**) Data set C, and (**d**) Data set D.

Figure 16 shows that ALSCN can almost completely identify the various health states of the bearings on the four data sets. The failure to achieve 100% accurate identification is because faults with different diameters at the same location may have an evolutionary relationship. Moreover, it is difficult for ALSCN to fully identify them. However, based on the current diagnostic accuracy rate, ALSCN can meet the requirements of industrial applications.

**Figure 17:**
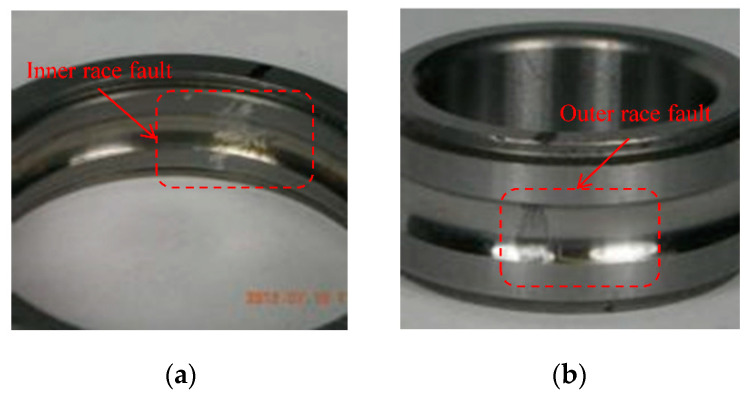
The two bearings from MFPT data sets: (**a**) inner race fault (IRF); (**b**) outer race fault (ORF).

In addition, the MFPT bearing fault data set [42] was also selected to conduct experiments to better verify the generalization of the network. The test stand is equipped with NICE bearings with a roller diameter is 0.235 inch, a pitch diameter is 1.245 inch, and a contact angle is 0. The two bearings with inner race fault (IRF) and outer race fault (ORF) are shown in Figure 17. Table 9 shows the settings of the MFPT data set training set and test set.

**Figure 18:**
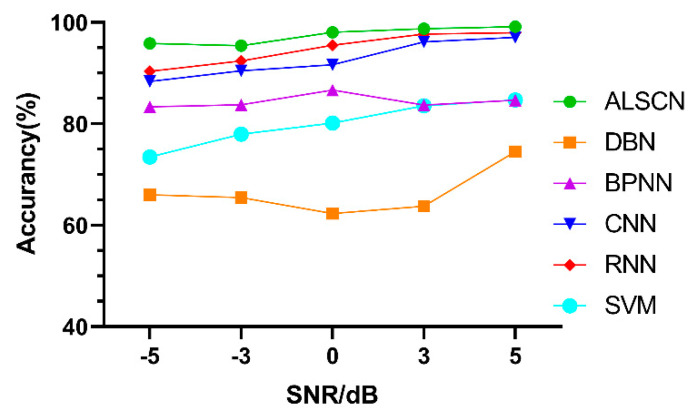
Test accuracy of different models under different SNR on MFPT.

Test accuracy of different models under different SNR on MFPT are shown in Figure 18 and Table 10. Although RNN and CNN have obtained high accuracy on the MFPT data set, the ALSCN still leads under five kinds of SNR conditions. 

## 5. Conclusions

An end-to-end ALSCN model is proposed to solve the problem that the accuracy of traditional and deep learning models decreased on longer signal under noise. Firstly, the fault diagnosis method based on ALSCN removes the process of manually extracting features. Secondly, it can adaptively adjust hyperparameters for different application conditions. Furthermore, the article developed two improved multi-scale modules to greatly preserve the spatial correlation on longer signals and make up the loss of detailed information under strong noise. This enables ALSCN to efficiently diagnose longer time series under strong noise of practical engineering application. The proposed model is validated for the robustness and generalization ability of the algorithm on CWRU and MFPT data set. The diagnosis results are compared with the traditional method SVM and deep learning methods CNN, DBN, BPNN, and RNN. The average accuracy of the ALSCN is improved by 9.34%, 12.27%, 24.17%, 22.59% and 42.02% on the four data sets of the CWRU, respectively. Under the strong noise (SNR = −5dB) on data set D, the accuracy of the ALSCN is increased by 8.69% compared to the well-performing CNN. On the MFPT data set, it also increased by 7.51% and 5.5%, respectively, compared with CNN and RNN. The method proposed in this paper has certain practical value and application prospects for the research of fault diagnosis based on long time series under noise.

In future work, author will explore the hardware implementation of this model and the real-time classification of more faults in rotating machinery.

## Figures and Tables

**Table 1:** Network process and output size of different stages.

Process		Output Size	Process		Output Size
Stage 0	Convolutional block	N × 20 × 5120	Stage 4	Convolutional block	N × 20 × 20
	4 × 1 max_pool	N × 20 × 1280		2 × 1 max_pool	N × 20 × 10
Stage 1	Multi_filter_layer	N × 40 × 1280	Stage 5	Convolutional block	N × 10 × 10
	Multi_pooling_layer	N × 40 × 320		2 × 1 max_pool	N × 10 × 5
Stage 2	Multi_filter_layer	N × 80 × 320	Stage 6	Convolutional block	N × 10 × 5
	Multi_pooling_layer	N × 80 × 80		5 × 1 max_pool	N × 7 × 5
Stage 3	Convolutional block	N × 40 × 80	Stage 7	Sigmoid	N × 7 × 1
	Multi_pooling_layer	N × 40 × 20			

**Table 2:** The main parameters of ALSCN.

	Kernel Number	Size	Stride
First convolutional layer	20	1	1
4 × 1 max pooling layer	/	4	4
Multi-filter layer	200	/	/
Multi-pooling layer	/	/	/
Second Multi-filter layer	400	/	/
Second Multi-pooling layer	/	/	/
Second convolutional layer	40	1	1
Third Multi-pooling layer	/	/	/
Third convolutional layer	20	1	1
2 × 1 pooling layer	/	2	2
Forth convolutional layer	10	1	1
2 × 1 pooling layer	/	2	2
Fifth convolutional layer	7	1	1
5 × 1 pooling layer	/	5	5
sigmoid	/	/	/

**Table 3:** Training set and Test set based on 10-fold cross-validation method of CWRU.

Data Set	Motor Speed/rpm	Load/hp	Number of Training Samples	Number of Test Samples	Fault Type
A	1772	1	530	133	NORMAL IF18 RF18 OF18 IF36 RF36 OF36
B	1750	2	530	133	
C	1730	3	530	133	
D	1797, 1772, 1750, 1730	0, 1, 2, 3	1590	399	

Note: NORMAL means the normal state. IF18, RF18, and OF18 indicate the inner ring fault, roller fault, and outer ring fault at 6:00 position relative to the load center, all with a fault diameter of 0.18mm. IF36, RF36, and OF36, respectively represent inner ring faults, roller faults, and outer ring faults at 6:00 position relative to the load center, all with a fault diameter of 0.36 mm.

**Table 4:** Comparison of the accuracy of different length signals based on convolutional neural networks (CNN).

	1024Dim Test (%)	5120Dim Test (%)
A	95.00	93.23
B	97.05	79.70
C	97.43	90.23
D	97.51	82.22
Average (%)	96.75	86.35

**Table 5:** Fault diagnosis results of the three models with different module orders.

	Model 1		Model 2		Model 3	
	Module 1	Module 2	Module 1	Module 2	Module 1	Module 2
Stage 0						
Stage 1	√	√	√		√	
Stage 2	√	√	√		√	
Stage 3		√		√	√	√
Stage 4				√		√
Stage 5				√		
Stage 6						
Stage 7						
Sigmoid						
Accuracy (%)	99.17		95.82		97.03	

Note: Module 1 is the multi-scale feature extraction module, and Module 2 is the multi-scale max pooling module.

**Table 6:** Fault diagnosis results of pooling layers with different orders.

Stage	Order 1	Order 2	Order 3
Stage 4	2 × 1	2 × 1	5 × 1
Stage 5	2 × 1	5 × 1	2 × 1
Stage 6	5 × 1	2 × 1	2 × 1
Accuracy (%)	99.17	96.76	95.07

**Table 7:** The accuracy of different models with different SNR value on CWRU.

SNR/(dB)	ALSCN	DBN	BPNN	CNN	RNN	SVM
−5	90.51 ± 1.09	68.45 ± 2.41	76.60 ± 0.64	81.82 ± 0.45	67.47 ± 3.52	70.11 ± 0.89
−3	92.53 ± 0.76	75.36 ± 1.99	75.51 ± 1.38	87.07 ± 0.92	80.61 ± 1.97	74.48 ± 1.27
0	93.74 ± 0.34	77.49 ± 2.08	76.41 ± 1.67	88.45 ± 1.03	95.15 ± 0.85	78.26 ± 0.33
3	98.99 ± 1.01	67.48 ± 3.44	76.57 ± 2.06	92.12 ± 0.14	97.78 ± 0.39	83.14 ± 1.64
5	99.07 ± 0.11	83.37 ± 1.78	75.36 ± 2.52	92.53 ± 0.57	97.95 ± 0.77	84.20 ± 1.17

**Table 8:** The accuracy of different models with different data sets on CWRU.

Data Sets	ALSCN	CNN	DBN	BPNN	RNN	SVM
A	98.18 ± 0.59	93.23 ± 0.82	74.51 ± 1.67	76.95 ± 2.14	39.10 ± 2.75	90.25 ± 0.37
B	98.05 ± 0.74	79.70 ± 1.96	70.06 ± 2.54	75.04 ± 1.62	38.35 ± 2.16	85.46 ± 0.94
C	99.11 ± 0.12	90.23 ± 1.20	75.65 ± 1.83	77.14 ± 1.99	51.35 ± 3.22	91.23 ± 0.47
D	99.12 ± 0.05	82.22 ± 0.81	77.57 ± 1.08	75.00 ± 1.47	97.59 ± 0.45	90.17 ± 1.03

**Table 9:** Training set and Testing set of mechanical fault prevention technology (MFPT).

Type of Fault	Load/1bs	Number of Training Samples	Number of Test Samples	Sampling Rate/sps
NORMAL	270	273	69	97,656
ORF1	270	273	69	97,656
ORF2	25, 50, 100, 150, 200, 250, 300	156	40	48,828
IRF	0, 50, 100, 150, 200, 250, 300	156	40	48,828

**Table 10:** The accuracy of different models with different SNR value on MFPT.

SNR/(dB)	ALSCN	DBN	BPNN	CNN	RNN	SVM
−5	95.87 ± 0.64	66.05 ± 3.12	83.39 ± 1.01	88.36 ± 0.32	90.37 ± 0.50	73.45 ± 1.25
−3	95.4 ± 0.24	65.45 ± 2.23	83.77 ± 0.92	90.45 ± 0.56	92.41 ± 0.37	77.98 ± 1.41
0	98.08 ± 0.92	62.32 ± 2.42	86.69 ± 1.01	91.66 ± 1.15	95.54 ± 0.22	80.17 ± 0.44
3	98.76 ± 0.23	63.76 ± 1.97	83.70 ± 1.74	96.17 ± 0.32	97.72 ± 0.87	83.60 ± 0.97
5	99.17 ± 0.03	74.52 ± 1.06	84.67 ± 1.49	97.06 ± 0.29	97.98 ± 0.34	84.73 ± 0.31
